# Potentially inappropriate prescriptions for older patients in long-term care

**DOI:** 10.1186/1471-2318-4-9

**Published:** 2004-10-15

**Authors:** Carol Rancourt, Jocelyne Moisan, Lucie Baillargeon, René Verreault, Danielle Laurin, Jean-Pierre Grégoire

**Affiliations:** 1 Health Economics and Outcomes Research, Merck Frosst Canada Ltd, Montreal, Qc, H9H 3L1, Canada; 2 Population Health Research Unit and Faculty of Pharmacy, Université Laval, Hôpital St-Sacrement, 1050 Chemin Ste-Foy, Québec, Qc, G1S 4L8, Canada; 3 Family Medicine Unit, Centre hospitalier universitaire de Québec, 2701 boul. Laurier, Québec, Qc, G1V 4G2, Canada; 4 Geriatric Research Unit, and Faculty of Medicine, Université Laval, Hôpital St-Sacrement, 1050 Chemin Ste-Foy Québec, Qc, G1S 4L8, Canada; 5 Geriatric Research Unit and Faculty of Pharmacy, Université Laval, Hôpital St-Sacrement, 1050 Chemin Ste-Foy Québec, Qc, G1S 4L8, Canada

## Abstract

**Background:**

Inappropriate medication use is a major healthcare issue for the elderly population. This study explored the prevalence of potentially inappropriate prescriptions (PIPs) in long-term care in metropolitan Quebec.

**Methods:**

A cross sectional chart review of 2,633 long-term care older patients of the Quebec City area was performed. An explicit criteria list for PIPs was developed based on the literature and validated by a modified Delphi method. Medication orders were reviewed to describe prescribing patterns and to determine the prevalence of PIPs. A multivariate analysis was performed to identify predictors of PIPs.

**Results:**

Almost all residents (94.0%) were receiving one or more prescribed medication; on average patients had 4.8 prescribed medications. A majority (54.7%) of treated patients had a potentially inappropriate prescription (PIP). Most common PIPs were drug interactions (33.9% of treated patients), followed by potentially inappropriate duration (23.6%), potentially inappropriate medication (14.7%) and potentially inappropriate dosage (9.6%). PIPs were most frequent for medications of the central nervous system (10.8% of prescribed medication). The likelihood of PIP increased significantly as the number of drugs prescribed increased (odds ratio [OR]: 1.38, 95% confidence interval [CI]: 1.33 – 1.43) and with the length of stay (OR: 1.78, CI: 1.43 – 2.20). On the other hand, the risk of receiving a PIP decreased with age.

**Conclusion:**

Potentially inappropriate prescribing is a serious problem in the highly medicated long-term care population in metropolitan Quebec. Use of explicit criteria lists may help identify the most critical issues and prioritize interventions to improve quality of care and patient safety.

## Background

Inappropriate medication use is a major health care issue for the elderly population [1-3]. Older patients are more at risk for adverse medication outcomes because they often have complex drug regimens and because of the age-related changes in drug pharmacokinetics and pharmacodynamics [1]. Potentially inappropriate prescriptions (PIPs), defined as prescriptions in which risks outweigh benefits, have been assessed in various settings using lists of explicit criteria most often based on that developed by Beers [4]. PIPs have been estimated to affect 4.8% to 45.6% of the elderly population [5-12].

Prevalence estimates of PIPs are likely to vary with the criteria that are applied. Some authors have based their assessment on the Beers criteria [5-7,9-12]. However, in all these studies but one [7], criteria applied were a subset only of Beers criteria as dosage and duration was not evaluated. Despite controversy about which explicit criteria should be used, there is a strong body of evidence showing that suboptimal prescribing is disturbingly common in older patients.

In Canada, a list of explicit criteria was developed by a panel of experts in 1997 [13]. The Canadian criteria required diagnostic information which is not easily accessible in the long-term care setting [6,14]. Using various methodologies, several studies have investigated the extent of the problem in Canada. A 1995 study of community-dwelling and institutionalized older patients reported large variations in PIPs among provinces, ranging from 4.8% in the prairies to 12.8% in Quebec [9]. More recently, the prevalence of PIPs in long-term care patients in Ontario was reported to range between 14.9% and 20.8% [15-17]. In Quebec, a 1990 retrospective database survey of 63,268 older Medicare patients reported that 45.6% of non-institutionalized patients received high-risk prescriptions of questionable appropriateness [8], while a recent survey of 3,400 elderly patients in the Quebec general population reported that 6.5% had a potentially inappropriate prescription (PIP) [18]. A 1995 physician survey reported that 77.1% of nursing home patients in Quebec had been taking benzodiazepine for over one year [19].

The long-term care elderly population is particularly vulnerable to inappropriate medication use; it is composed of frail older patients who typically have functional disabilities and acute and chronic medical histories that require complex medication regimens [20,21]. Assessing PIPs using the data available in long-term care, in particular data on dosage and duration of use, may help designing efficient interventions to improve prescribing practices in one of the frailest populations. The objectives of this study were (1) to describe prescribing patterns in elderly patients residing in long-term care facilities in the Quebec metropolitan area, (2) to assess the prevalence of PIPs in this long-term care setting using published explicit criteria [4,13,22] adapted for this study, and (3) to identify patient-related predictors of PIPs.

## Methods

### Design and data sources

A cross-sectional chart review of long-term care patients aged 65 years and over living in the Quebec City area was performed in the period between April 1995 and December 1996. All long-term care facilities located in the Quebec City area were contacted and the majority (29 out of 33) agreed to participate in the study. Within the 29 participating facilities, there were a total of 71 long-term care units. Numbers of beds in these units averaged 41 (10 to 190). Units were visited once during the study period. Data on drugs currently being prescribed the day of the visit was collected using medication charts. Demographic data included age, gender and length of stay. This study was approved by the ethics committees at Université Laval, Hôpital Saint-François d'Assise and Hôpital de l'Enfant-Jésus.

For each medication order, the name, dosage, frequency of dosing and nature of prescription (scheduled or given on an as-needed basis) were collected. To capture the fullest possible extent of potentially inappropriate prescribing, it was assumed that all medications prescribed on an as-needed basis were taken. The total daily dose of an as-needed prescription was calculated by multiplying the prescribed unit dose with the indicated daily frequency of administration. Prescriptions for creams, ointments and drops were not included. Each medication was classified using the Anatomical Therapeutic Chemical (ATC) classification system [23]. The maximal prescribed daily dose was calculated for each medication order.

### Classification of potentially inappropriate prescribing

A list of explicit criteria for PIP in older patients was developed based on a review of the literature [4,6,10,11,13,14,22]. Criteria referring to medications unavailable in Canada were excluded. Because diagnostic information is difficult to obtain in the long-term care setting [6,14], criteria involving clinical information were also excluded. The list of criteria was elaborated using a modified Delphi method [24]. A consensus panel of four local experts was convened including a general practitioner with a geriatric practice (RV), a family physician (LB), a clinical pharmacist and a pharmacoepidemiologist (JPG), all involved in practice or research on medication issues in the elderly population. In the first step, experts were asked to review and comment independently on the preliminary list of published criteria. Responses from the experts were used to revise this list. In the second step, the panel discussed each criterion until a consensus was reached. A total of 111 explicit criteria were included in the list to assess the quality of prescribing (Appendix).

Medication charts were reviewed and compared with the list of explicit criteria. PIPs were categorized as:

• Potentially inappropriate medication;

• Potentially inappropriate duration;

• Potentially inappropriate dosage; and

• Potentially inappropriate drug-drug interaction.

### Data analyses

Drug prescribed and PIP data were stratified by age and gender. Chi-square and Student *t *tests were used to compare proportions and means, respectively. Association between age and drug utilization was evaluated by analysis of variance. Factors predicting PIP were identified by logistic regression analyses. Independent variables were age, sex, number of prescribed drugs and length of stay. An initial bivariate analysis allowed calculation of crude odds ratios, identification of variables individually associated with the risk of PIP, and determination of the appropriate scale for each variable. A multivariate analysis with a significance threshold of 0.10 for the inclusion of variables subsequently yielded adjusted odds ratios for the number of prescribed medications, age and length of stay. Data were analyzed for collinearity and overdispersion. Data analyses were performed using SAS version 6.12 (SAS Institute Inc. Cary, NC).

## Results

### Study population

The study population included 2,633 individuals, aged 65 years and older, residing in long-term care facilities for a mean duration of 8.5 years. Mean age was 82 ± 8 years and the majority of individuals were women (74.2%). Women were older than men (84 ± 8 years versus 79 ± 8 years, *p *= .0001).

### Drug utilization

Most residents (94%, n = 2,481) had one or more prescribed medications and 48% (n = 1,266) of the total population had five or more medications. Residents had on average 4.8 prescribed medications. Proportions of patients by number of prescribed medications were similar for men and women, but varied according to age. The oldest patients, aged 85 years and more, received significantly less medications than their youngest counterparts aged between 65 and 74 years; 43.8% of patients aged over 85 years received five medications or more, compared to 59.4% of those aged 65 to 74 years. Of the 12,707 medications prescribed, 86% were scheduled administrations and 82% were prescribed for more than three months.

A majority of patients (85.5%, n = 2,251 patients) had a prescription for medications of the central nervous system (CNS). Cardiovascular medications (46.4%, n = 1,221 patients) and medications of the alimentary tract and metabolism (29.3%, n = 772 patients) were the following most frequently prescribed anatomical groups of medications. Most commonly prescribed therapeutic classes included analgesics (48.0%), anxiolytics (41.4%), antipsychotics (35.0%) and loop (high-ceiling) diuretics (18.6%) (Table 1). There were differences in therapeutic classes prescribed to men and women. Acetaminophen (36.7% of patients), haloperidol (20.5% of patients) and lorazepam (20.2% of patients) were the three most frequently prescribed drugs (Table 2).

**Table 1:** Proportion (in %) of elderly patients on medication by therapeutic class and sex*

	**Proportion of patients (%)**			
	
**Therapeutic class**	**All (n = 2,633)**	**Men (n = 680)**	**Women (n = 1,953)**	***p *value**
Analgesics & antipyretic	48.0	44.6	49.2	0.037
Anxyolitics	41.4	41.4	41.4	0.983
Antipsychotics	35.0	39.5	33.5	0.004
Loop (high ceiling) diuretics	18.6	16.2	19.4	0.062
Antiepileptics	14.9	21.6	12.6	<0.001
Thyroid preparations	14.6	8.4	16.8	<0.001
Vasodilators	14.6	11.0	15.8	0.002
Antidepressants	13.7	11.0	14.7	0.017
Cardiac glycosides	12.4	11.6	12.7	0.462
Drugs for peptic ulcer	11.0	11.3	10.9	0.765
Hypnotics & sedatives	10.9	9.8	11.3	0.293
Anticholinergics	10.9	11.2	10.8	0.788
Selective calcium channel blockers	10.6	7.2	11.7	0.001
Angiotensin converting enzyme inhibitors	10.0	10.0	10.0	0.991

* Only therapeutic classes prescribed to 10% or more of the elderly are displayed

**Table 2:** Most frequently prescribed medications among the elderly in long-term care

		**Proportion of patients (%)**		
		
**ATC code**	**Medication**	**Men (n = 680)**	**Women (n = 1,953)**	**All (n = 2,633)**
N02BE01	Acetaminophen	30.5	38.9	36.7
N05AD01	Haloperidol	21.9	20.1	20.5
N05BA06	Lorazepam	20.2	20.2	20.2
C03CA01	Furosemide	16.2	19.2	18.6
N02BA01	Acetyl salicylic acid	19.1	16.7	17.3
N05BA04	Oxazepam	16.3	17.1	16.9
H03AA01	Levothyroxin sodium	8.4	16.8	14.6
C01DA02	Nitroglycerin	10.0	14.6	13.4
C01AA05	Digoxin	11.6	12.7	12.4

ATC: Anatomical Therapeutic Classification

### Potentially inappropriate prescribing

Overall, 51.5% of the population under study had one or more PIPs. Of the 2,481 patients with at least one prescribed drug, more than half (54.7%) had one or more PIPs; 29.5% had one PIP, 12.5% had two PIPs, 7.5% had three PIPs and 5.3% had four or more PIPs.

A total of 12,707 drugs were prescribed of which 1807 were given on an as-needed basis. The proportion of PIPs among scheduled and as-needed prescriptions were 9.2% and 11.5%, respectively. If we exclude as-needed prescriptions, 46.4% of all residents had one or more PIPs.

**Figure 1:**
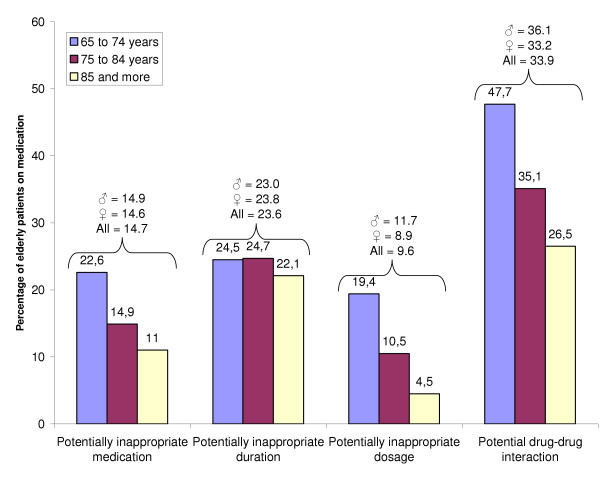
Potentially inappropriate prescribing including inappropriate medication, dosage, duration and potential drug-drug interaction among three age-groupsof long-term care elderly (N = 2,481) ♂ = male ♀ = female

The most common type of PIP was drug-drug interaction, affecting 33.9% of patients treated with drugs, followed by potentially inappropriate duration (23.6%), potentially inappropriate medication (14.7%), and potentially inappropriate dosage (9.6%) (Figure 1). The proportion of patients receiving any type of PIP decreased with age, from 66.7% for patients aged 65 to 74 years to 56.4% for those aged 75 to 84 years and 47.7% for patients aged 85 years and more. PIPs were the most frequent for CNS medications, representing 9.3% of prescribed medications.

The most common PIP was a potentially inappropriate duration for intermediate and short-acting benzodiazepines for more than one month (22.9%); more than half of those PIPs were for the anxiolytic oxazepam (Table 3). A substantial number of patients treated with pharmacotherapy were receiving repeat (dual) prescriptions of antipsychotics (16.5%) or benzodiazepines (14.9%). Almost 6% of patients treated with pharmacotherapy were prescribed potentially inappropriate long-acting benzodiazepines and 5.2% were receiving haloperidol at a potentially inappropriate dosage. The most common PIP among cardiovascular drugs was repeat prescription of calcium channel blockers, affecting 3.1% of treated patients.

**Table 3:** Most common potentially inappropriate prescriptions (PIPs) among older patients receiving medication in long-term care

**Criteria**	**Number of patients**	**Proportion of all patients prescribed a medication (%) (N = 2,481)**
**Potentially inappropriate medication**	**365**	**14.7**
Long-acting benzodiazepines	138	5.6
Preparations including an antihistaminic	112	4.5
Flurazepam	54	2.2
Doxepin	31	1.3
Amitryptiline	27	1.1
Propanolol	27	1.1
Chloral hydrate	22	0.9
**Potentially inappropriate duration**	**585**	**23.6**
Intermediate and short-acting benzodiazepines at bedtime for more than one month	567	22.9
Oxazepam at bedtime for more than one month	313	12.6
**Potentially inappropriate dosage**	**239**	**9.6**
Haloperidol > 3 mg daily	129	5.2
Thioridazine > 30 mg daily	53	2.1
Lorazepam > 3 mg daily	34	1.4
**Potential drug-drug interaction**	**842**	**33.9**
Repeat* prescription of antipsychotics	409	16.5
Repeat* prescription of benzodiazepine	369	14.9
Clonazepam and other benzodiazepine	46	1.9
Benzodiazepine and hypnotic or sedative	93	3.8
Repeat* prescription of calcium channel blockers	77	3.1
Repeat* prescription of tricyclic antidepressants	37	1.5
Repeat* prescription of angiotensin converting enzyme inhibitors	19	0.8
Repeat* prescription of β-blockers	11	0.4
Repeat* prescription of non-steroidal anti-inflammatory drugs (except acetylsalicylic acid)	10	0.4
Repeat* prescription of barbiturate	10	0.4
Total potential inappropriate prescriptions**	**1,358**	**54.7**

*Repeat prescription indicates that two agents of the same drug class are being prescribed

**Numbers do not add up since one prescription may be linked to more than one PIP (e.g., duration and dosage)

### Predictors

Multivariate analysis indicated that patients with a length of stay 10 years or over were 1.78 times at greater risk of being prescribed a PIP than those with less than 10 years of stay (adjusted odds ratio [OR]: 1.78, 95% confidence interval [CI]: 1.43–2.20) (Table 4). The risk of PIP also increased significantly as the number of drugs prescribed increased (OR: 1.36, CI: 1.32–1.41) whereas it decreased with age. Gender was not a significant predictor of PIP. No problems of collinearity or overdispersion were observed in the multivariate model.

**Table 4:** Predictors of potentially inappropriate prescription among elderly patients in long-term care (N = 2,481)

**Predictor**	**Proportion of patients with PIP (%)**	**Crude odds ratio (95% CI)**	Adjusted odds ratio (95% CI)*
Number of prescribed drugs (increments of one drug)	54.7	1.38 (1.33–1.43)	1.36 (1.32–1.41)
Gender			
Women	54.5	1.00	-
Men	55.5	1.04 (0.87–1.25)	-
Age			
65 to 74 years	66.7	1.00	1.00
75 to 84 years	56.4	0.65 (0.51–0.81)	0.74 (0.58–0.96)
85 years or more	47.7	0.46 (0.36–0.57)	0.60 (0.47–0.77)
Length of stay			
<10 years	51.1	1.00	1.00
≥10 years	67.4	1.98 (1.62–2.41)	1.78 (1.43–2.20)

CI: confidence interval; *Adjusted for number of prescriptions, age, and length of stay

## Discussion

The long-term care elderly population evaluated in this study was highly medicated and a majority of patients receiving medication had a PIP. These results indicate that potentially inappropriate prescribing was significant at the time of the study in institutionalized older patients in the Quebec metropolitan area.

A total of 94% of residents in this long-term care population were prescribed at least one drug, compared to 60% in community-dwelling elderly patients in Quebec [25]. The mean number of medications was also higher (4.8) than in community-dwelling individuals in Quebec (2.9) [25], but lower than in American long-term care (7.2) [7].

The total prevalence of PIPs among the population under study was high (51.5%). Estimates of PIP prevalence in the literature vary between 4.8% [6] and 45.6% [8] for both institutionalized and community-dwelling older patients. Caution must be used when comparing these results, as the delivery of care may vary from one setting and one region to another [9]. The current lack of consensus when defining lists of criteria and variations with respect to methodologies also contribute to the observed differences [26]. For example, Zhan and colleagues [5] estimated the proportion of potentially inappropriate medication use in the community-dwelling elderly in the United States. Applying criteria on the indication for the use of 33 drugs, they observed a prevalence of 21.3% for 1996. In our study, PIPs were identified using an explicit criteria list that was primarily based on Beers and McLeod criteria [4,13,22] and that was updated and validated by local experts to apply to the long-term care context in Quebec. As we had access to dosage and duration information, we were able to apply a broader set of criteria which can explain the higher prevalence of PIPs we have observed. Explicit criteria lists, such as those developed by Beers and McLeod, define inappropriate prescription according to the drug overall risk-benefit profile for elderly patients. These lists were previously used in studies examining inappropriate prescribing in elderly populations [3,5,6,11,15,27-30] and undergo a continuous process of revision and updating to reflect the most current clinical information on the risks and benefits of medications [31].

A large number of patients were receiving CNS medication (85%) and the most common PIPs were related to that category of drugs. Thirty-five percent of patients were prescribed antipsychotics and 22.9% had benzodiazepine for potentially inappropriate duration, defined as more than a month [32]. A number of studies have reported the inappropriate use of CNS drugs [5,8,33,34], particularly benzodiazepines [18,19]. Many factors may contribute to the continued use of inappropriate CNS medications, including prescriber attitudes, patient demands and the design of the health care system [34]. A survey of physicians in Quebec reported that the psychological distress of aging patients and the quasi-absence of reported side-effects justified the long-term use of psychotropic medication, which was seen as the most effective way of helping the patient [35]. Moreover, side effects of psychoactive medication are often believed to be a consequence of the aging process [34]. Almost three quarters of potentially inappropriate psychoactive medications can produce a physical dependence [34]. Psychoactive pharmacotherapy increases risk of hip fractures and is advocated for use with caution to prevent falls in elderly populations [36,37]. Anticonvulsants, antidepressants and short- and long-acting benzodiazepines were reported to increase risk of falls in older women [38].

The length of stay was positively associated with PIPs, while the prevalence of PIPs decreased with age. Although the association between length of stay and the likelihood of receiving a PIP in nursing homes was studied in the past [6], to our knowledge, this is the first time it is being shown to be a predictor of PIPs. On the other hand, the risk of receiving a PIP was previously reported to decrease with age in nursing home patients over 65 years [7,12]. Data on clinical status was not considered in these studies and it can be hypothesized that either the oldest residents were less ill or that physicians were more cautious when prescribing to very old patients. As reported in previous studies [12,26], the number of medications was also a predictor of PIP in older patients. Patients in long-term care frequently have multiple diseases resulting in complex medication regimens, which makes assessment of the risks versus benefits of treatments often difficult. Female gender was previously reported as a predictor of PIP [7,12]. Although we observed gender differences in the prescribed therapeutic classes, female gender was not a predictor of PIPs in our study.

The results presented here should be viewed in light of potential limitations. As in previous studies [15], we did not abstract information on diagnoses from the patients charts and drug prescriptions were considered as surrogates for disease conditions. Thus, the explicit criteria used in this study apply to general circumstances, but may not be applicable to specific cases, since they do not consider clinical information. For example, lipid-lowering drugs may be potentially inappropriate in patients aged 75 and over, but evidence from clinical trials suggests that statins may be of benefit if the patient's life expectancy exceeds two years [39]. Thus, misidentification of potential cases of appropriate or inappropriate prescribing may have occurred, since complex medical conditions can alter the risk-benefit profile of medications. However, due to the frail condition of most patients, it is unlikely that such misidentifications have occurred frequently. Since access to clinical data is often difficult in the nursing home setting, a list of explicit criteria that does not require that type of information may be easier to apply on a larger scale.

This study evaluated prescription patterns rather than the actual consumption of medication. The low prevalence of as-needed medication (14%) and the long-term care setting, in which medication is administered to patients by a health caregiver, suggest that this limitation did not have a significant impact on the results. As-needed prescriptions may have accounted for repeat prescriptions, which may in turn have led to overestimation of the number of drug-drug interactions. However, even after excluding as-needed prescriptions from the analysis, the proportion of residents with a PIP remains high.

Predictors of PIPs were assessed using a multivariate analysis. It allowed us to adjust for potential confounding variables. However, we were not able to adjust for facility variables as those were not available.

This study is the first to describe and qualify prescribing practices in long-term care facilities in urban Quebec. In particular, it highlights the extent of potentially inappropriate prescribing in elderly long-term care patients, which are among the frailest of society [4,21]. Inappropriate prescribing is one component of the major health care problem of suboptimal prescribing that also includes underuse of effective agents, drug-disease interactions and prescription errors. Substantial morbidity, mortality and cost are attributed to suboptimal prescribing [1,2]. Although a decline in the prevalence of PIPs was reported in community-dwelling older patients in the United States between 1987 and 1996 [40], the continued use of inappropriate medications is a major concern. A growing body of evidence suggests that clinical pharmacy and multidisciplinary team interventions can modify suboptimal prescribing in older patients. Modern data management [15,41] and use of the best clinical evidence could help practitioners improve the management of complex cases [40,42]. Recent studies in long-term care settings showed that physician or pharmacist interventions reduce PIPs [1,12,16,43], while a clinical review program of prescriptions for community-dwelling patients conducted by a team of physicians, pharmacists and nurses did not seem to improve prescribing practices [44].

## Conclusions

Inappropriate prescribing is highly prevalent in the elderly long-term care population in metropolitan Quebec. The use of a explicit criteria list to identify PIPs is a first step towards identifying most critical issues and implementing strategies to improve quality of care and patient safety. Identifying predictors of PIPs may help to target problems and prioritize interventions that are most needed in the rapidly expanding older population.

## Competing interests

Carol Rancourt and Jean-Pierre Grégoire were employed by Merck Frosst Canada at the time of the preparation of this article.

## Authors' contributions

CR, in partial fulfillment for the grade of M.Sc., lead the protocol development, expert panel consultation, data analyses, discussion of results, and manuscript preparation. JM contributed to all steps of this research project and manuscript preparation. LB contributed to protocol development, presentation and discussion of results and manuscript preparation and participated in the expert panel to define the explicit criteria. RV is the principal investigator for the initial research project which generated the drug prescription data used for this study. He contributed to protocol development, presentation and discussion of results, manuscript preparation and participated in the expert panel to define the explicit criteria. DL was a co-investigator for the initial research project, which generated the drug prescription data used for this study, and contributed to protocol development, data analyses and manuscript preparation. All authors read and approved the final manuscript. JPG contributed to protocol development presentation and discussion of results, manuscript preparation and participated in the expert panel to define the explicit criteria.

## Pre-publication history

The pre-publication history for this paper can be accessed here:



## Supplementary Material

Additional File 1 Appendix: List of explicit criteria used to assess the quality of prescribing in long-term care for elderly patients provide as additional file Click here for file
